# Photo-responsive liquid crystal network-based material with adaptive modulus for haptic application

**DOI:** 10.1038/s41598-022-24106-8

**Published:** 2022-11-14

**Authors:** Ievgen Kurylo, Joost van der Tol, Nicholas Colonnese, Dirk J. Broer, Danqing Liu

**Affiliations:** 1 Laboratory of Stimuli-Responsive Functional Materials and Devices (SFD), Department of Chemical Engineering and Chemistry, Eindhoven University of Technology, Den Dolech 2, 5612 AZ Eindhoven, The Netherlands; 2 Laboratory of Macro-Organic Chemistry, Department of Chemical Engineering and Chemistry, Eindhoven University of Technology, Den Dolech 2, 5612 AZ Eindhoven, The Netherlands; 3 Meta Reality Labs Research, Redmond, WA USA

**Keywords:** Materials for devices, Soft materials

## Abstract

Artificially created tactile feedback is in high demand due to fast developments in robotics, remote control in medicine, virtual reality, and smart electronics. Despite significant progress, high-quality haptic feedback devices remain challenging mainly due to the lack of stability and spatiotemporal resolution. In this work, we address these issues by the application of dynamic coatings, based on photo-responsive liquid crystal network (LCN) material. This material adapts upon an external stimulus (UV light with a power intensity of 50–90 mW/cm^2^) that changes its elastic properties (87% decrease of the modulus for 90 mW/cm^2^ power intensity of 365 nm UV light). Localized change of adaptive modulus with very high resolution (2 μm) was demonstrated.

Touch feeling belongs to the 5 human senses and it is implemented via force/position and pressure receptors of the skin, related to manipulation, perception, and communication. It helps us to interact with, and navigate through the environment (physical or virtual). Touch supplies information about shape, hardness, roughness, texture, and temperature via direct contact with an object.

Artificially created sense of touch or tactile feedback is an area of haptic technology or kinesthetic communication^1^. Demand for such technologies is appearing due to fast developments in robotics, remote control in medicine, virtual reality, and smart electronics^2,3^.

Materials and devices for haptic application of such technologies as mechanical vibration^4^, responsive polymers^5,6^ microelectromechanical system (MEMS)^7^, Peltier elements^8^, lasers^9^, ultrasound^10^, pneumatics^11^, air jets^12^, surface acoustic waves^13^, electrostatics^14^ etc.

Despite significant progress, high-quality haptic feedback devices remain challenging mainly due to the lack of stability and spatiotemporal resolution. One way to address these issues is the application of dynamic coatings. They adapt upon an external stimulus that changes their properties and provide on-screen tactile feedback at the position of touch by localizing it with a high resolution (micrometers) in various perception forms, such as surfaces that change their topography^15^ or switch between “dry” and ‘’wet’’^16^. Direct change of the material provides a real rather than simulated sensation of touch.

In this work we study such dynamic coatings with adaptive modulus based on a liquid crystal network (LCN)^17,18^, which is a widely studied material for active surfaces^19^. It permits predefining, freezing and subsequent tuning the molecular order, which, in turn, influences the macroscopic properties of the material^20–22^. Low molar mass liquid crystal (LC) acrylates^23^ are an attractive choice as building blocks for LCNs, as they are easy to orient and more applicable for coatings, especially for those with complex geometries, due to their low processing viscosity. As soon as the desired molecular alignment is established during the coating process, the LC monomers are cured by photopolymerization fixing the molecular organization in a network structure. Triggered manipulation of the molecular order changes the polymer properties such as wettability, elastic modulus, glass transition temperature, density etc. Artificially created adaptive modulus materials^24^ are known and based on molecular rearrangement upon application of an external stimulus such as heat, light, or electricity. LCN-based photoactive materials are intensively studied^25–32^. Almost invariably, their photo-responsiveness relies on the usage of azobenzene-containing molecules^33^ either covalently incorporated into the network structure or as guest additive. These examples comprise a shape-memory LCN-based material capable of dynamic modulus change upon photoactivation, previously developed by our group^34^. Such effect was obtained due to the incorporation of a low amount (2 wt%) of azobenzene-containing diacrylate in the LCN structure. Reversible isomerization of this photo-responsive agent, triggered by light with wavelengths close to azobenzene stereoisomers absorption maximum, ~ 365 nm (*trans* to *cis*) and ~ 450 nm (*cis* to *trans*), leads to molecular oscillations, distortion of the molecular order, free volume generation and, finally, photo-softening^35^. Excess free volume is energetically unfavorable^15^, thus the effect disappears when an external stimulus is removed. Modulus change can be amplified by dual-wavelength illumination^34^, which leads, however to substantial overheating and is less convenient for practical usage. One potential way to overcome this issue is to incorporate a small amount of fluorescent dye^36^. It emits photons with wavelength within *cis*-azobenzene absorption band after excitation with 365 nm light which was explored for temporal free volume generation^37^.

In the present study, we demonstrate a strong amplification (280% for a coating on a glass surface) of modulus change due to the incorporation of a fluorescent dye in the material structure. We also demonstrate the translation of the adaptive modulus effect from a free-standing film to a coating and localize it in a designed location of the sample. We envision that this unique approach enables the enhanced performance of haptic communication devices due to the possibility of fine-tuning the elasticity change (via UV power intensity control) and complex haptic information communication due to very high resolution (~ 2 μm). We believe that our findings permit a significant step toward the real-life application of haptic technology as such coatings can be applied to modify objects, enabling them with a haptic communication feature. Finally, the localization of modulus change on designed locations on the surface is essential for the communication of spatially resolved complex haptic information.

## Results and discussion

### Changing LCN-based material properties via incorporation of fluorescent dye

We prepared a LCN, with the composition shown in Fig. 6, in the presence and absence of fluorescent compound 7 to determine its influence on the isomerization reaction of the azobenzene moiety. We have chosen a material with a chiral-nematic molecular order. Due to the helicoidal organization of the mesogens with the helix axis orthogonal to the film/coating surface, an in-plane isodeformation occurs upon a change of the molecular order. Consequently, the azobenzene moieties are predominantly aligned with their transition moment in the plane of the film/coating, which enhances their absorption of incoming light.

**Figure 1:**
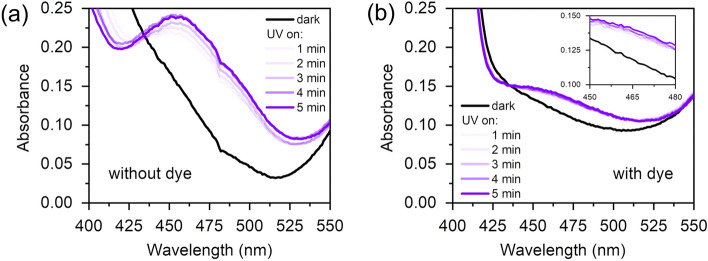
Absorption spectra of 6 μm thick LCN films without (**a**) and with dye (**b**) in dark and after UV illumination with 1–5 min duration. Power is ~ 50 mW/cm^2^.

**Figure 2:**
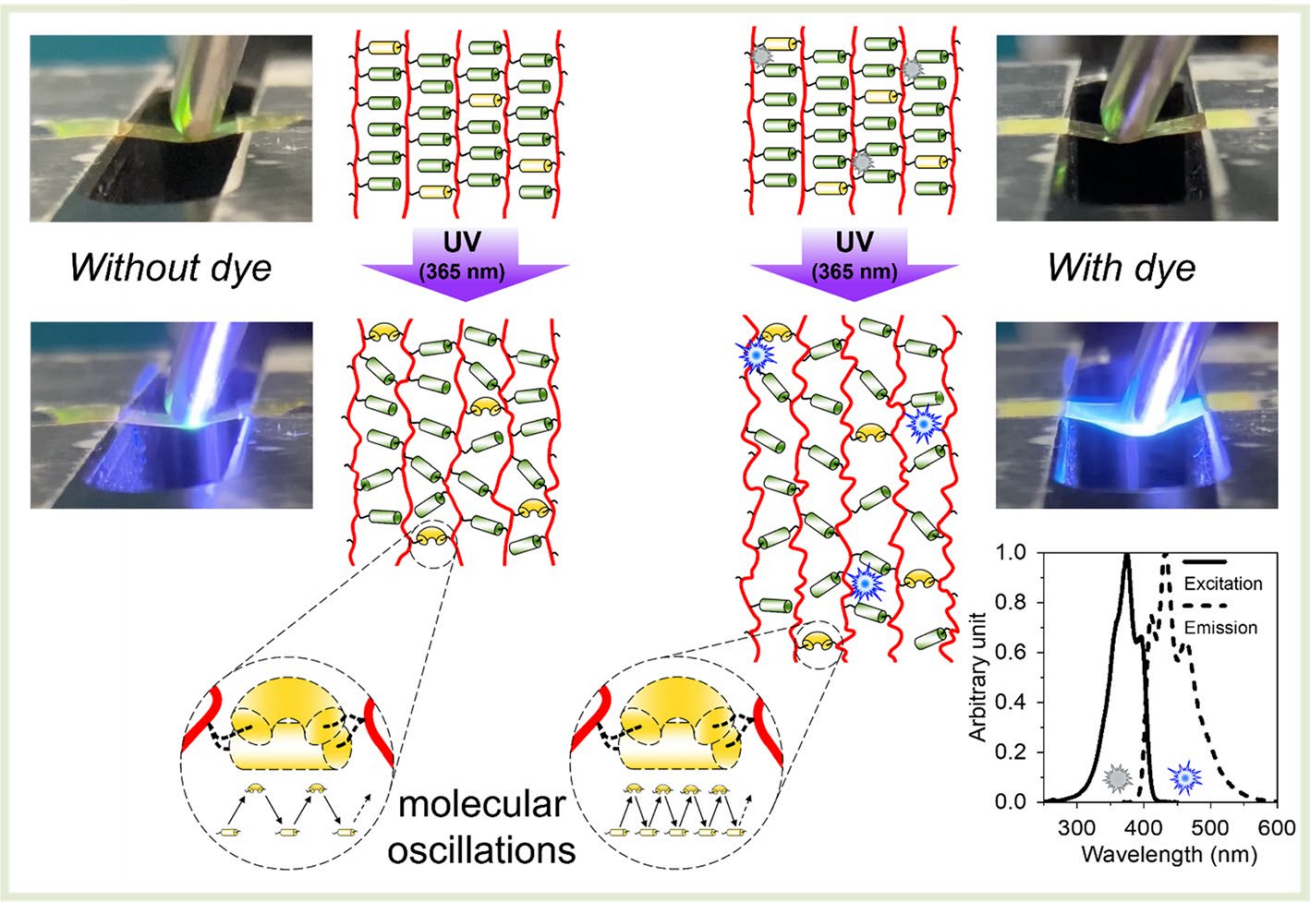
Amplification of photo-softening effect by incorporation of blue-emitting fluorescent dye (excitation and emission spectra are shown) in LCN structure. LCN films, containing azobenzene units (in yellow), connected from both sides to polyacrylate chains (in red) remain rigid in a normal state. Upon UV illumination of dye-free film, azobenzene units are predominantly in the *cis* state. The low oscillation frequency induces the moderate formation of free volume and softening of the material. During UV illumination of the dye-containing film, additional blue light emission results in simultaneous triggering of both the forward and backward isomerization reactions. This leads to a higher frequency of molecular oscillations and induces a larger free volume increase and change in material elasticity. For clarity, helicoidal structure of LCN is not shown. See supplementary video 1 for a real-life demonstration of the changing mechanical properties of the dye-containing material.

Absorbance spectra, recorded before and after UV illumination are shown in Fig. 1. As the fluorescent dye is strongly absorbing below 400 nm (see Fig. 2), we did not analyze the *trans* azobenzene absorbance region. The data indicate that the *trans* to *cis* conversion for the dye-free LCN film is 4.8 times larger than for the sample with dye. Hence, there is coexistence of considerable amounts of the *trans* and *cis* azobenzene-containing mesogens at equilibrium conditions (reached within 5 min).

The coexistence of *trans* and *cis* azobenzene is an indication of intense oscillatory transitions between stereoisomers induced by combined irradiation with UV (from LED) and blue light (from fluorescent dye). Indeed, this reaction is reversible and, consequently, should be considered as a dynamic process. It leads to a stronger plasticizing effect of polyacrylate chains and amplification of the photo-softening effect (Fig. 2).

Importantly, only a small fraction (~ 2%) of 365 nm light is transmitted through the sample coating at equilibrium for the dye-containing sample, while for the dye-free sample it is as high as ~ 20% (Fig. S1 and supplementary video 2). These observations signify that the dye-containing LCN film, placed in between the light source and human skin, in a real-life application drastically reduces the harmful impact of UV irradiation.

### Amplification of bulk modulus change

**Figure 3:**
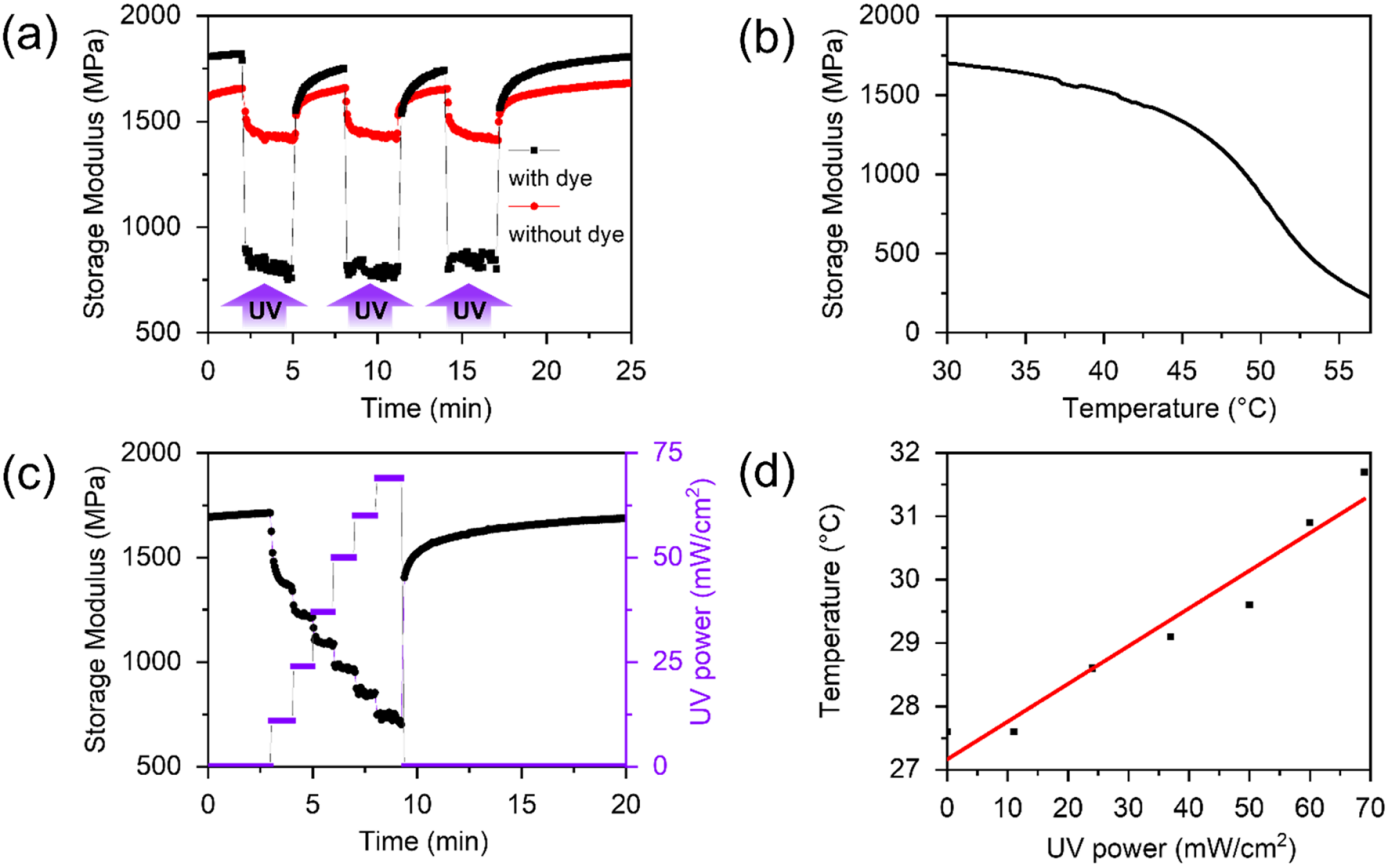
Storage modulus of chiral-nematic LCN films with and without fluorescent dye under UV illumination with 3 min intervals and power ~ 50 mW/cm^2^ (**a**). Modulus-temperature relation (without illumination) for the identical sample (**b**). Storage modulus change of dye-containing chiral nematic LCN film using different UV power intensities (**c**) and corresponding temperature changes (d).

We performed dynamic mechanical analysis on the samples with and without the fluorescent dye. In the absence of an external stimulus their elasticities are similar and in the range of 1.6–1.8 GPa at room temperature (Fig. 3a). Under UV illumination a clear contrast in elastic properties for the samples was observed. Specifically, for the dye-containing sample the modulus decreases by ~ 970 MPa. For dye-free sample this effect is more moderate: a decrease of ~ 230 MPa is observed. Hence, the addition of fluorescent dye leads to significant amplification of the modulus change in line with our initial assumption. Both response time and relaxation time (restoring initially high modulus value) are within 15 s indicating that mechanical equilibrium is reached faster than photochemical equilibrium. Sequential photoactivation leads to a repetitive identical decrease of the modulus for both samples.

Temperature-related modulus changes are minor (Fig. 3b) for the investigated power range. Sample illumination with the sequentially increasing power of UV light leads to a rising storage modulus change in line with UV intensity (Fig. 3c). Relative change of the modulus is most significant for the first illumination event which is related to the relaxation of initially present stresses in the polymer structure. The temperature rises linearly with irradiation intensity (Fig. 3d). Linear changes in temperature and modulus as functions of power permit prediction and precise control of the material elasticity by will.

### Translating adaptive modulus from free-standing film to a coating

**Figure 4:**
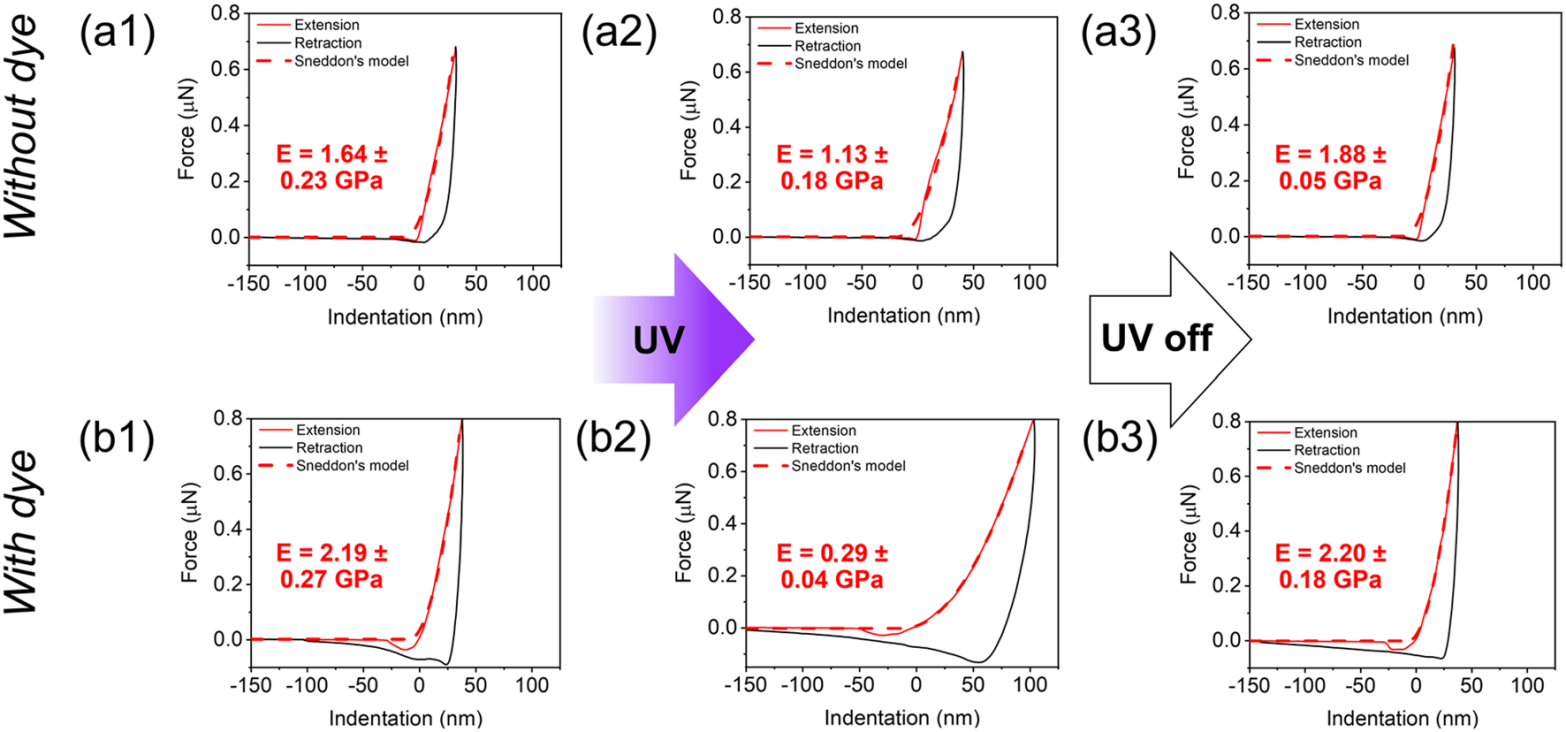
Force curves of chiral-nematic LCN coatings without dye (**a1**–**a3**) and with dye (**b1**–**b3**) on a glass substrate in dark (**a1** and **b1**), under UV illumination with power ~ 90 mW/cm^2^ (**a2** and **b2**) and 2 min after switching off UV light (**a3** and **b3**).

The prospect of transferring the concept of reversible modulus change from bulk material to a coating was studied via AFM. For this purpose, force-indentation curves of LCN coatings on glass surfaces with the same composition as for bulk modulus measurements were recorded. Identical measurements were conducted for the sample with and without dye in dark, during UV illumination (~ 90 mW/cm^2^), and after switching off UV light (Fig. 4).

For the same applied force (stress), an increase in tip indentation (strain) during UV light is observed for both samples. After illumination, the indentation recovers back to its initial level. These data reflect the evolution of elastic modulus as it is inversely proportional to strain. It is also an indication of the photo-softening effect on the investigated surfaces. Modulus values calculated from these data prove substantial amplification of elasticity reduction by the presence of the fluorescent dye in the LCN coating. Indeed, modulus decreased by 31% and 87% for the samples without and with dye respectively. Accordingly, we have demonstrated 280% amplification of modulus change due to the presence of the fluorescent dye in the material. Such modulus change (from 2.2 to 0.3 GPa) can be sensed by human^37^. The temperature during illumination is estimated to be 38 °C and 33 °C for the samples with and without dye respectively, which proves that modulus change occurs due to the photo-softening effect.

Remarkably, we observed a significant modulus change regardless of the direction of applied force. Indeed, during bulk modulus measurements using DMTA, the free-standing LCN film was stretched perpendicularly to the helix director. While for AFM experiments, the force was applied parallel to the helix axis (perpendicularly to the surface). Such tolerance to the direction of the applied force can be explained by the concept of free volume. It is known for LCNs that during photoactivation the intermolecular spaces increase^38^. This enables plasticized polyacrylate main chains to be deformed in any direction.

UV penetration depth could become an issue for the thicker samples (due to the strong absorbance of trans-azobenzene and the fluorescencent dye). However, similar changes of the modulus upon illumination for the dye-containing film were observed by AFM and DMTA signifying that the UV light reaches throughout the entire sample.

The increasing lag between retraction and extension curves during photoactivation indicates amplification of adhesion upon illumination. For the dye-containing sample, this effect is much more pronounced (at 0.2 µN the lag is 45.2 nm and 25.7 nm for the sample with and without dye respectively). Indeed, a lower modulus increases the indentation depth and therefore the contact area between the AFM tip leading to higher adhesive forces and adhesive work.

### Localization modulus change

**Figure 5:**
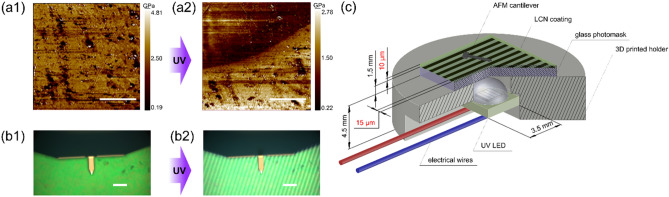
Modulus distribution on dye-containing LCN film before (**a1**) and during (**a2**) UV illumination with the photomask. Corresponding AFM camera images (**b1** and **b2**), showing the AFM cantilever and the photomask pattern, which appears under UV illumination. Schematic representation of the set-up used (**c**). Dimensions of the LCN coating and the photomask are depicted in red. Scale bars: 10 μm (**a1** and **a2**) and 100 μm (**b1** and **b2**).

To enable complex and instructive haptic information transfer, patterned modulus change is a prerequisite. We, therefore, studied localized softening effects by means of AFM via recording a force map under UV illumination. By using a photomask containing 15 µm linear patterns (Fig. 5c), we could guarantee the presence of illuminated and “dark” areas (Fig. 5) and measure corresponding modulus values for a 25 × 25 µm region of the coating surface. The modulus of the same area was recorded before and during illumination (Fig. 5a).

A clear contrast with a gradual transition of ~ 2 µm between low and high modulus zones was observed in coherence with illuminated and dark regions of the sample (Fig. 5a2). The same area, recorded in dark has a homogeneously distributed high modulus zone. These data indicate a spatially restricted photo-softening effect with high (micrometer scale) resolution. Hence, by tuning the size and shape of photomask patterns, desired haptic information can theoretically be communicated.

It should be noted, that despite the clear contrast between low and high modulus, the average modulus values of the sample region that is covered by the mask during the exposure procedure are lower than those measured for the samples in absence of any irradiation. This is related to partial illumination of these regions due to diffraction, scattering, and light reflection from the cantilever surface (which is in nanometer-scale proximity to the analyzed spot).

The difference in absolute modulus values recorded for force mapping (Fig. 5) and single force (Fig. 4) experiments is related to high (125 s^-1^) and low (0.25 s^-1^) strain rates applied respectively, as elastic modulus is higher for larger strain rates^39^.

## Conclusions

In the present study, we developed adaptive modulus surfaces based on chiral-nematic LCN. The material is significantly photo-softened (87% decrease of the modulus for a coating on glass) by bringing covalently connected azobenzene-containing moieties into an oscillating mode by exposure with low-intensity UV light (~ 90 mW/cm^2^) triggering *trans* to *cis* isomerization. The backward, *cis* to *trans,* reaction is simultaneously triggered by additional exposure to blue light, due to the presence of a small amount (0.5%) of fluorescent dye avoiding the use of two light sources. Due to a substantial amount of *trans* isomer at the thermodynamic equilibrium, only a small fraction of 365 nm light (~ 2% for a 10 μm sample) is transmitted, which reduces the harmful effect of UV light upon human contact. Moreover, higher energy efficiency and minor overheating were achieved in comparison to previously studied dual-wavelength illumination of dye-free samples. In addition, we have demonstrated successful localization of modulus change with a high resolution (around 2 μm). We envision that our original approach enables the fabrication of haptic devices with enhanced performance in the near future.

## Materials and methods

### Materials

**Figure 6:**
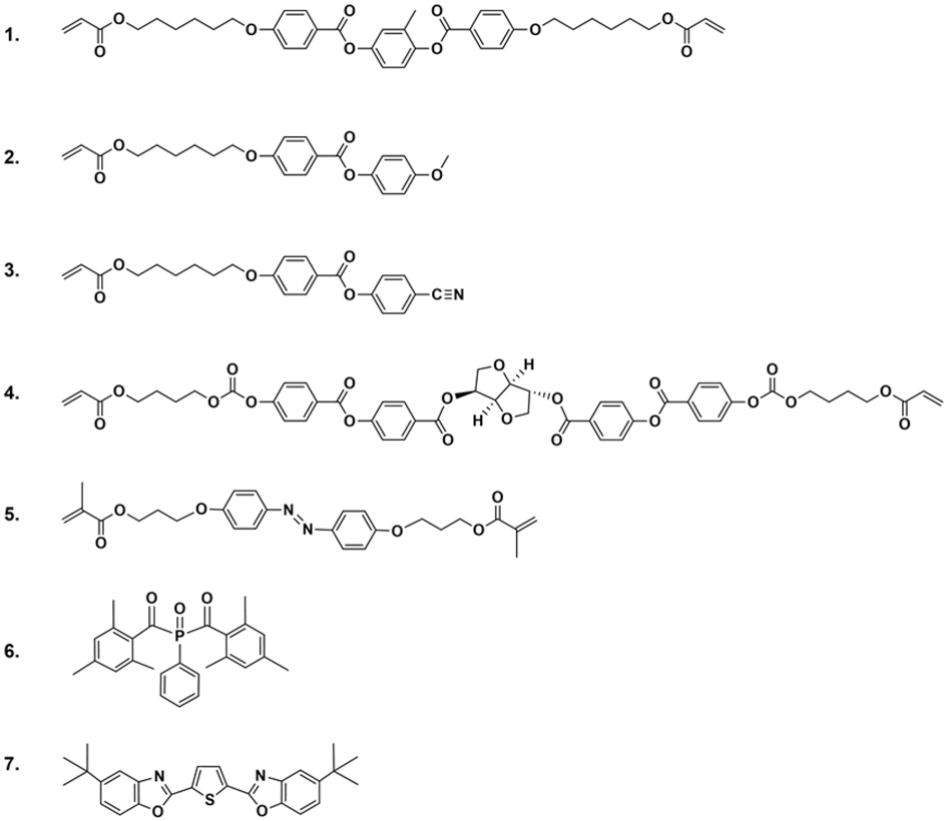
Applied materials. Liquid crystal monomers (1–3), polymerizable chiral dopant (4), polymerizable azobenzene (5), photoinitiator (6), fluorescent dye (7).

Figure 6 shows the mixture used to produce an adaptive modulus LCN material.

Monomers 1 to 3 were obtained from Merck UK. Monomer 4 was obtained from BASF. Monomer 5 was custom-synthesized by Syncom (Groningen, the Netherlands). Photoinitiator 6 was purchased from Ciba Specialty Chemicals. Fluorescent dye 7, 2,5-bis (5-tertbutyl-benzoxazol-2-yl) thiophene, was obtained from Sigma Aldrich. Polymer films and coatings were fabricated using a mixture containing 21.9 (or 22.4 for the reference sample) wt% monomer 1, 40.6 wt% monomer 2, 31 wt% monomer 3, 4 wt% monomer 4, 2 wt% monomer 5, 1 wt% photoinitiator 6 and 0.5 (or 0 for the reference sample) wt% fluorescent dye 7. The constituents were mixed by dissolving in dichloromethane.

### Sample preparation

Glass substrates are cleaned by a 5-min dip in acetone and 2-propanol sequentially while stirring followed by flushing with demi water and drying with a nitrogen flow. A1051 (Sunever, Nissan Chemical, Japan) was used to obtain a planar alignment of the liquid crystal monomer mixture. It was spin coated on cleaned glass followed by baking at oven for 90 min at 180 °C. Gentle manual rubbing on a velvet cloth gave the substrate the surface structure needed for desired LC alignment. A cell provided with alignment layers was capillary filled with the LC monomer mixture and subsequently cured by UV exposure at 42 °C for 30 min with an intensity of 400 mW/cm^2^ using a mercury lamp (EXPR Omnicure S2000) equipped with a cut-off filter transmitting light > 400 nm to prevent premature isomerization of the azobenzene group during polymerization. The samples were post-cured at 120 °C to ensure full cure of the acrylate monomers.

### Sample characterization

UV-Vis spectroscopy was performed on a LAMBDA™ 750 UV/Vis/NIR spectrophotometer (PerkinElmer) equipped with a 150 mm integrating sphere. Sample films with a thickness of 6 um were illuminated with 365 nm LED (Mouser Electronics) between measurements with intensity ~ 50 mW/cm^2^ and 1–5 min duration.

The macroscopic mechanical properties of the films were acquired using dynamic mechanical thermal analysis (DMTA) (Q800 Dynamic Mechanical Analyzer from TA Instruments) at a frequency of 1 Hz. For this, a sample of 10 × 3 × 0.01 mm dimensions was fixed at both ends in clamps and characterized in a strain-controlled mode. 365 nm LED (Mouser Electronics) is used to provide monochromatic light with power is ~ 50 mW/cm^2^. Temperature was monitored remotely using an infrared camera (Fluke Ti-32).

Atomic force microscopy images and force measurements were recorded in tapping and contact mode, respectively, using a Cypher Environmental Scanner (ES) equipped with a closed cell and a normal sample stage. Force measurements were carried out using a super luminescent diode in order to reduce the signal-to-noise ratio. Silicon (100) AC160TS(-R3) probes (Oxford Instruments, spring constant k = 8.77–29.51 N/m) were used for all measurements and calibrated using the ‘Get Real’ function in the Igor Pro software. It should be noted that spring constants below 20 N/m were desirable for proper measurements. 365 nm LED (Mouser Electronics) embedded in home-made 3D printed holder was used to provide monochromatic light with power ~ 90 mW/cm^2^. Temperature was monitored remotely by reproducing experimental conditions outside the AFM compartment using an infrared camera (Fluke Ti-32). Processing of the images were done to enhance the contrast using plane fit and flattening at order 1 using Gwyddion v2.60. For the AFM force spectroscopy (AFM-FS), force-distance curves were recorded in a 20 × 20 µm area by approaching and retracting the cantilever tip to the sample on various spots before, during and after illumination with 365 nm light at a constant velocity of 1 µm/s. Additional force maps of 30 × 30 µm (128 × 128 pixels) were recorded before and during illumination with 365 nm light at a scan rate of 0.39 Hz and a Z-rate of 125 Hz. All AFM measurements were acquired in air and at the machine’s temperature (~ 30 °C). For our AFM force-distance curves and maps, we employed Sneddon’s model for cone-shaped AFM tips in order to fit the experimental data and obtain the elastic modulus of the sample:

$$F=\frac{2}{\pi }*\frac{{E}_{s}}{1-{V}_{s}^{2}}*\mathit{tan}\alpha *{\delta }^{2}(Eq.X)$$

where F is the applied force, E_s_ is the elastic modulus, V_s_ is the Poisson’s ratio and α the opening angle of the AFM tip and δ the indentation depth.

## Supplementary Information

Supplementary Video 1.

Supplementary Information 1.

Supplementary Video 2.

## Data Availability

The datasets generated and/or analysed during the current study are not publicly available due funding restrictions but are available from the corresponding author on reasonable request.
