# Supplementary material for: Photo-responsive liquid crystal network-based material with adaptive modulus for haptic application
Source: Sci Rep. 2022 Nov 14;12:19512. doi: 10.1038/s41598-022-24106-8 (PMC9663702; doi:10.1038/s41598-022-24106-8)
Supplement: Supplementary file 2 — Supplementary Information 1. [file 41598_2022_24106_MOESM2_ESM.docx]

Supplementary Figures


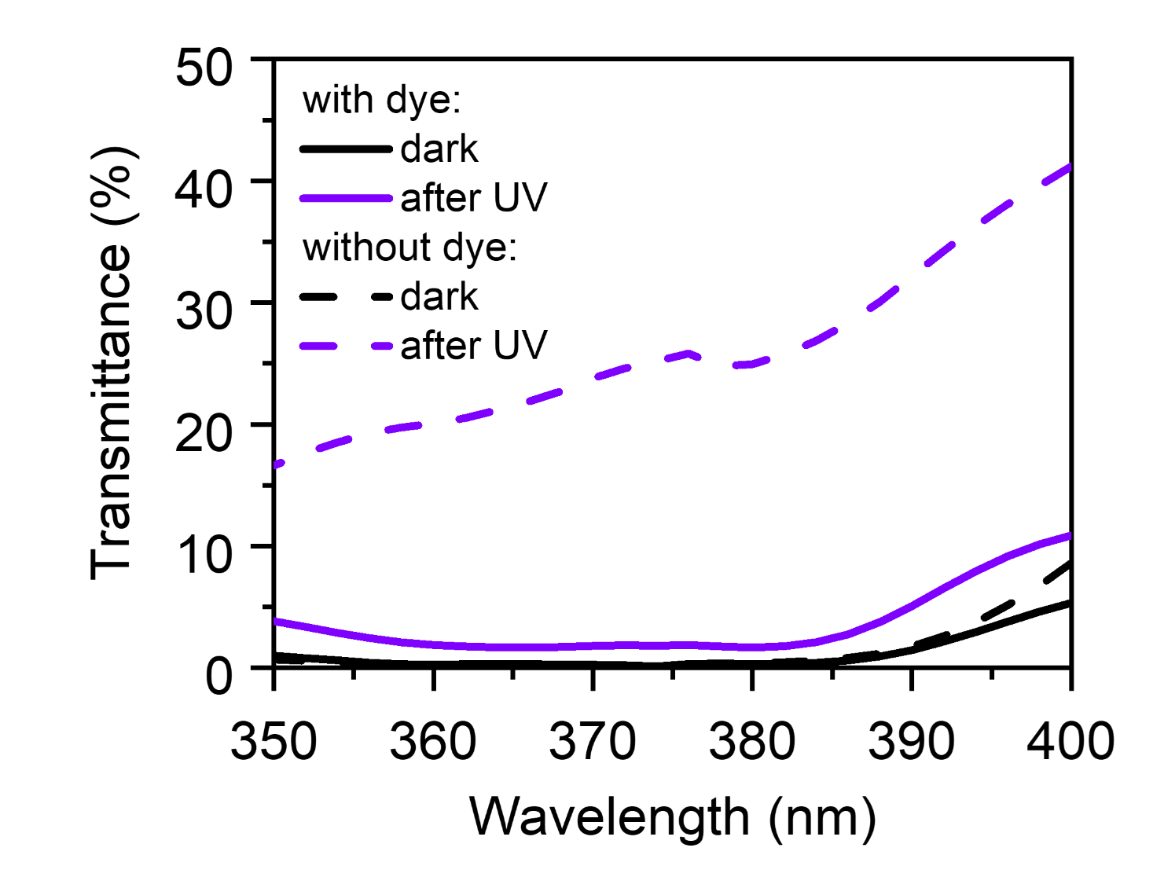


**Figure S1.** Transmittance spectra of 10 μm LCN coatings with and without dye in dark and after UV illumination (near-equilibrium conditions).


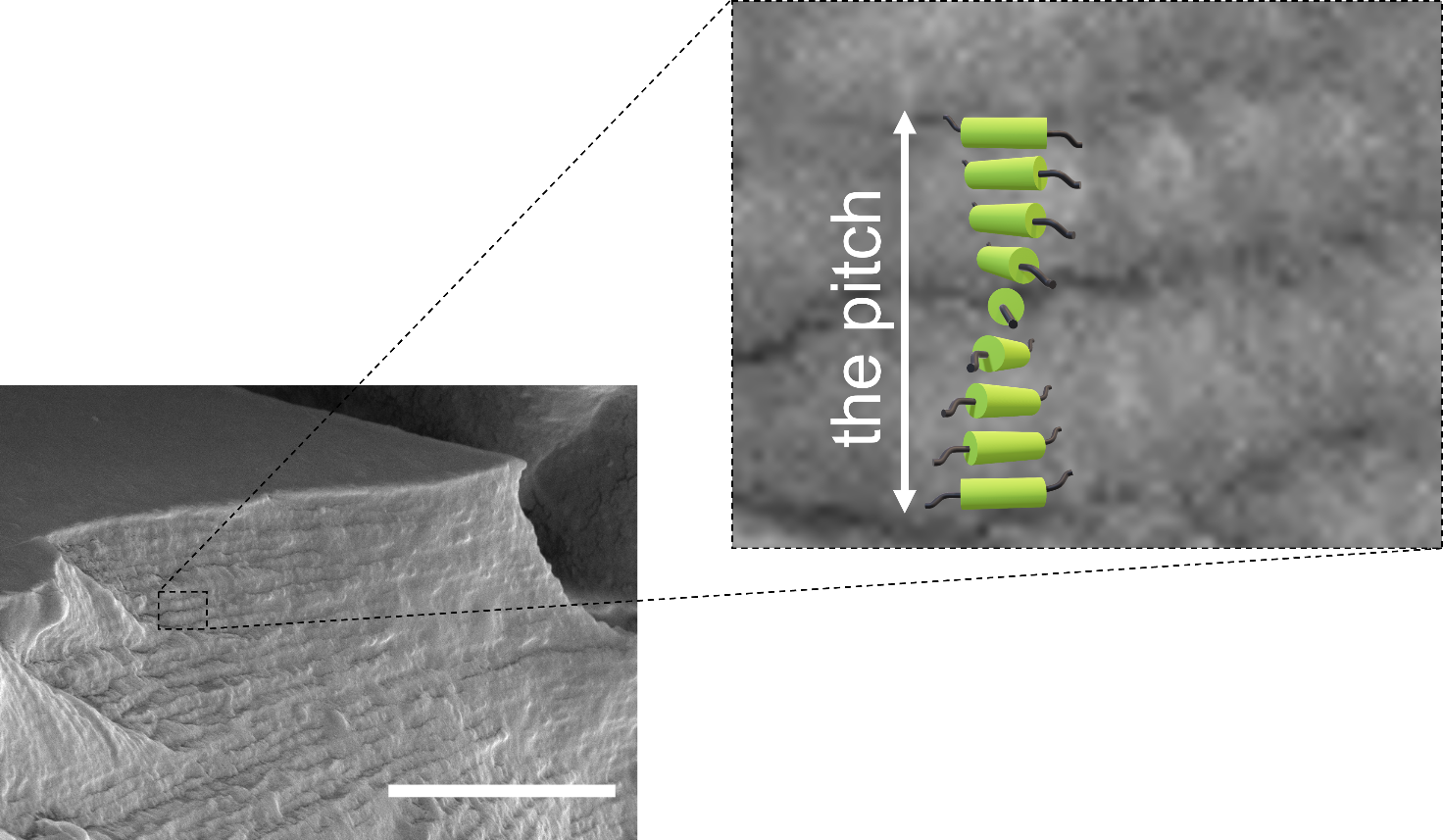


**Figure S2.** SEM image of a cross-section of the dye-containing LCN coating. Helicoidal pitch is pitch = 396 ± 40 (nm). Scale bar - 4 μm.


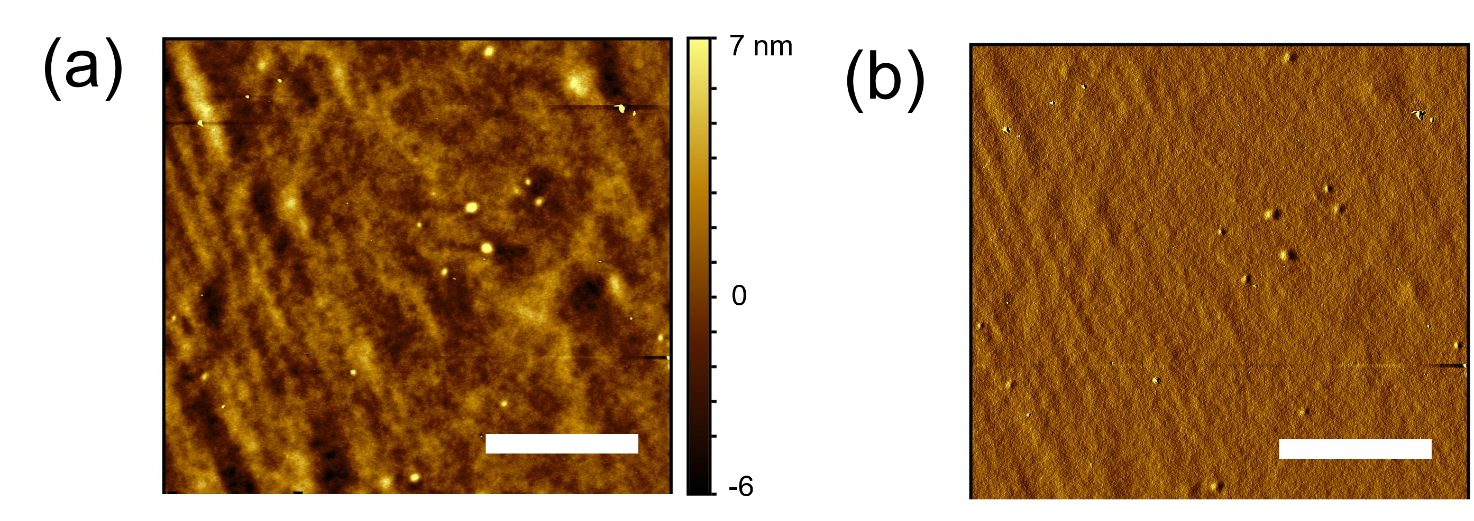


**Figure S3.** AFM images of height (a) and amplitude (b) profiles of the dye-containing LCN film. Scale bar - 5 μm.


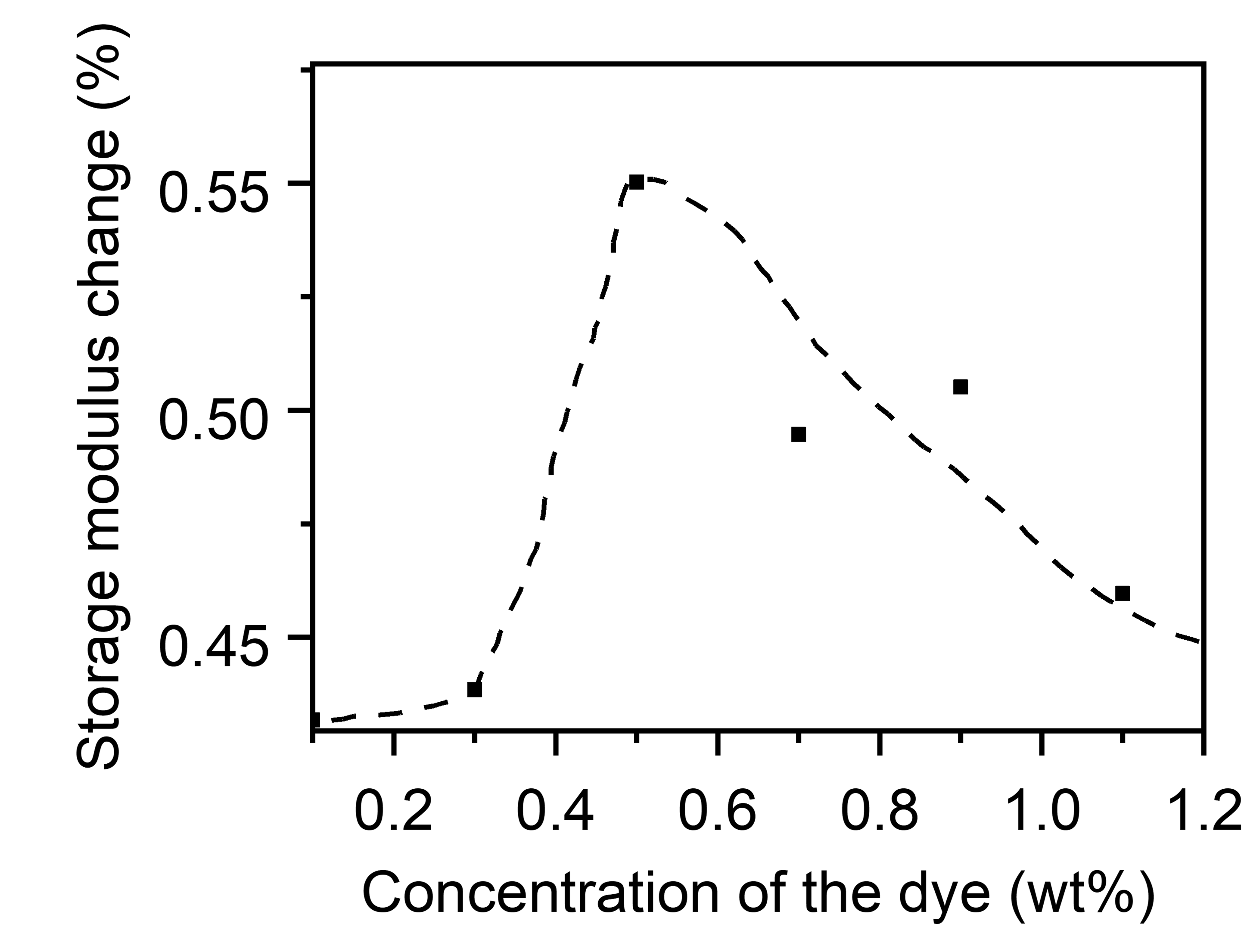


**Figure S4.** Storage modulus change (in %) of LCN films with different concentrations of dye. Thermal-related modulus change was taken into account for a more accurate comparison.


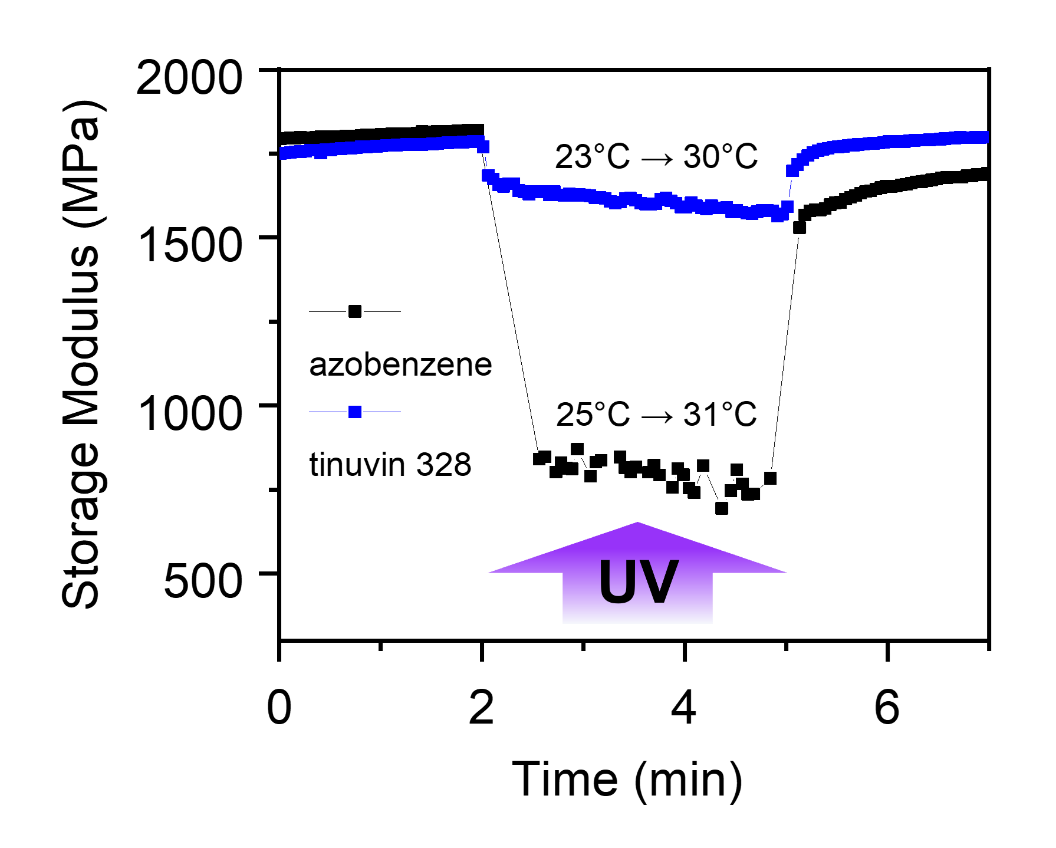


**Figure S5.** Modulus change under UV illumination (~50 mW/cm^2^) of dye-containing LCN film with normal composition and with azobenzene, replaced by UV absorbing molecule (tinuvin 328). Temperature change during illumination is indicated for both samples.
